# Psychometric properties of the person-centred coordinated care experience questionnaire (P3CEQ) in a Norwegian radiotherapy setting

**DOI:** 10.1093/intqhc/mzac067

**Published:** 2022-08-25

**Authors:** May ingvild volungholen Sollid, Marit Slaaen, Signe Danielsen, Øyvind Kirkevold

**Affiliations:** Research Centre for Age Related Functional Decline and Diseases, Innlandet Hospital Trust, Box 68, Ottestad 2313, Norway; Faculty of Medicine and Health Sciences, Department of Health Sciences, Norwegian University of Science and Technology (NTNU) Gjøvik, Box 191, Gjøvik 2802, Norway; Research Centre for Age Related Functional Decline and Diseases, Innlandet Hospital Trust, Box 68, Ottestad 2313, Norway; Institute of Clinical Medicine, Faculty of Medicine, University of Oslo, Box 1171, Blindern, Oslo 0318, Norway; Department of Oncology, St. Olav’s Hospital, Trondheim University Hospital, Box 3250 Torgarden, Trondheim 7006, Norway; Department of Physics, Norwegian University of Science and Technology (NTNU), Box 8900 Torgarden, Trondheim 7491, Norway; Research Centre for Age Related Functional Decline and Diseases, Innlandet Hospital Trust, Box 68, Ottestad 2313, Norway; Faculty of Medicine and Health Sciences, Department of Health Sciences, Norwegian University of Science and Technology (NTNU) Gjøvik, Box 191, Gjøvik 2802, Norway; Norwegian National Centre for Ageing and Health, Vestfold Hospital Trust, Box 2136, Tønsberg 3103, Norway

**Keywords:** patient experience, patient-reported experience measure, radiotherapy, quality improvement

## Abstract

**Background:**

The number of older adults with cancer is increasing. Radiotherapy is an important treatment modality for cancer and may cause side effects and distress. Patient-reported experience measures aim to measure patients’ experiences with health care. This can help healthcare services to improve in line with patients’ needs. To assess how Norwegian patients receiving radiotherapy experience their care, a valid and reliable tool is required. We selected the person-centred coordinated care experience questionnaire as a tool.

**Objective:**

The aim of the study is to validate the Norwegian version of the person-centred coordinated care experience questionnaire in a radiotherapy setting.

**Methods:**

A feasibility study of the person-centred coordinated care experience questionnaire and a cross-sectional study—testing psychometric properties of the questionnaire in a Norwegian radiotherapy setting—were conducted. Participants were recruited from two different hospitals in Norway. Patient characteristics and item scores are described using descriptive statistics. We performed an exploratory factor analysis and applied principal component analysis with a varimax rotation. Cronbach’s *α* was used to assess internal consistency.

**Results:**

In total, 24 patients participated in the feasibility test, and 176 were included in the cross-sectional study where we explored the psychometric properties of the person-centred coordinated care experience questionnaire. Three factors were identified. Internal consistency was established for the 10-item scale, with Cronbach’s *α* = 0.698.

**Conclusions:**

Conclusions must consider the Norwegian setting and healthcare context. We found that the Norwegian version of the person-centred coordinated care experience questionnaire is a relevant, valid and reliable tool to provide insight into different areas of patients’ experiences upon receiving radiotherapy. However, further testing on a larger sample is necessitated.

## Introduction

As life expectancy keeps increasing, so does the number of older adults with cancer [1]. The majority of cancer patients are ≥65 years [2]. Radiotherapy is required in ∼50% of cancer incident cases [3] and may be administered with curative or palliative intent. Patients receive radiotherapy as daily fractions, over days or weeks, most frequently as outpatient care. As cancer care requires multidisciplinary collaboration and coordination, services may be fragmented [4, 5]. Both cancer illness and radiotherapy may cause substantial distress, and patients’ experiences from radiotherapy can endure long after treatment concludes [6, 7]. This calls for efficient and high-quality services for a patient group with heterogeneous and complex needs.

The importance of factoring patients’ views and experiences into improvement and development processes in health care is well established, and patients’ experiences and users’ involvement in quality improvement are indispensable [8, 9]. Documented associations between clinical effectiveness and patient safety supports the inclusion of patient experiences in quality improvement, as well as in patient safety work [10]. Norwegian legislation has requested a system to register patients’ feedback in order to improve services in accordance with users’ needs [9].

International studies exploring perceptions of care in patients receiving radiotherapy find that patients experience high-quality care, but areas for improvement are recognized [11, 12]. These include needs for sufficient support and information before and during treatment [11, 12]. Good quality care should be respectful and responsive to patients’ preferences, needs and values [9]. To identify areas of importance for this treatment group, we conducted a qualitative study [13] and found that areas of importance for older patients with cancer receiving radiotherapy are information needs, involvement of next of kin, shared decision-making in treatment, and coordination of services [13]. This is in line with the person-centred care (PCC), which today is the state of the art in all treatment and care [14]. However, radiotherapy services have been criticized for not being patient-centred [11, 15].

To assess how Norwegian patients receiving radiotherapy experience their care, a valid and reliable tool is required [16]. Patient-reported experience measures (PREMs) are instruments aiming to measure the experience with health care received from the patient’s view [16]. International studies show increased use of PREMs to identify current areas of quality improvement, which allows healthcare services to improve in line with patients’ needs [10, 16, 17]. However, knowledge of how Norwegian patients receiving radiotherapy evaluate their care is limited, and there are seemingly no available PREMs to assess this. Some instruments are available for cancer care [18–20], but we have not managed to identify any appropriate measure to evaluate patients’ experiences with the entire radiotherapy trajectory. We therefore reviewed different PREMs to identify the most suitable tool. The results from the qualitative study [13] show that areas of importance to patients receiving radiotherapy are closely related to the domains in the person-centred coordinated care experience questionnaire (P3CEQ) [14, 21]. Thus, the P3CEQ was selected as a measure of patient experience in the current project. However, reassessing a measure’s reliability and validity to ensure tool performance is recommended when applying the measure in a new setting [16].

We performed a study aiming to adapt the P3CEQ to the Norwegian language and radiotherapy setting. Furthermore, we wanted to test the feasibility, face validity, construct validity and reliability of the Norwegian version of the P3CEQ.

## Methods

Our methods included a feasibility study (Step 1) of the selected questionnaire (i.e. P3CEQ) and a cross-sectional study (Step 2), testing the psychometric properties of the P3CEQ.

### Person-centred coordinated care experience questionnaire

The P3CEQ–English version has previously been psychometrically tested and validated among frequent users of general practice aged ≥18 years by Lloyd *et al*. [21]. The scale has been translated into Norwegian guided by principles for translation and cultural adaptation of patient-reported outcome measures [22–24], and developers provided us with the translated version. However, we have not been able to find published psychometric tests of the Norwegian version in a radiotherapy setting.

The P3CEQ is based on the theory of PCC and also focuses on experienced coordination of services [14, 21]. The instrument probes patients’ own goals or outcomes, care planning, care coordination, transitions, decision-making, information and communication. The instrument contains 10 main items, with one of the questions (Item 7a) triggering sub-questions concerning care plans (Items 7b–d). Additionally, there are two optional items concerning involvement of next of kin [21], both not included in the topical scale tests.

### Scoring

Eight items are Likert-scored [25], from 0 to 3, where higher scores indicate experiencing more person-centred coordinated care [21]. The response option ‘not relevant’ is scored 0 for all items. Each scoring option has appropriate explanatory text, see details in Table 3. Two items (6 and 7a) are scored dichotomously, either 0 or 3. A total score is calculated by summing all items. When calculating an overall score for the scale, developers recommend calculating an average of Items 7a–d [21]. The maximum score is 30, and the minimum score is 0. High scores indicate a high level of experienced person-centered coordinated care (PPC). Additionally, each single item has a comments section in order to allow individuals to elaborate their views in their own words [14]. These qualitative data will not be presented in this paper.

### Recruitment

Participants were recruited from radiotherapy units at a local hospital (Steps 1 and 2) and a university hospital (Step 2), in two separate Norwegian regions in November–December 2020 (Step 1) and January–September 2021 (Step 2). Patients were recruited in their final week of radiotherapy. Recruitment was done by a project nurse and the first author at the local hospital and by radiotherapists at the university hospital. We aimed for an even distribution among men and women, and age groups of ≥65 or ≤65 years.

### Inclusion and exclusion criteria

Our *inclusion criteria* comprised patients aged ≥18 years, with a confirmed cancer diagnosis, receiving radiotherapy with palliative or curative intent at one of the two hospitals. Any radiotherapy schedule and prescribed total dose was allowed. Patients had to be able to fill a self-report questionnaire and be fluent in Norwegian. The e*xclusion criteria* were being too fragile or ill to participate, as evaluated by an oncologist.

### Data collection

We distributed 30 questionnaires at the local hospital in Step 1 and 240 questionnaires—120 at each hospital—in Step 2 of the study.

Consenting participants received a questionnaire (P3CEQ) to be self-completed at home and returned in a pre-paid envelope. Self-completion at home was chosen to ensure that their answers truly reflected their experiences. The fill-in instructions inquired about the overall experience of care received, from radiotherapy referral until treatment concluded.

In addition, we collected socio-demographic characteristics such as gender, age, marital status, level of education and living arrangements. Health and treatment-related information (i.e. diagnosis, treatment aim and treatment metrics) were recorded from patients’ medical records.

Patients who declined participation were recorded by registering gender, age and general reasons for declining.

### User involvement

Two user representatives, one of the breast cancer society and one of the prostate cancer society, were consulted. These participated in the planning of the overall project and participated in the adjustments of the P3CEQ as part of evaluating the feasibility study.

### Statistical analysis

Statistical analysis was performed using IBM SPSS Statistics for Windows, Version 26.0. (IBM Corp, Armonk, NY, USA). Normally distributed values are reported with mean and standard deviation (SD), and skewed values with median and 25th and 75th percentiles (25pc and 75pc). Patient characteristics and item scores are described using descriptive statistics. Furthermore, we performed an exploratory factor analysis, and applied principal component analysis, with a varimax rotation [26, 27], as recommended in adapting measures to a new population, to evaluate construct validity [28]. Cronbach’s *α* was used to assess internal consistency to evaluate the reliability of the scale and sub-scales [29, 30].

It was desirable with a patient-to-item ratio of a minimum of 5:1, or preferably higher, to reduce the likelihood of errors of inference regarding the factor structure of the P3CEQ [26, 27]. With the number of items in the P3CEQ short form (10 items), the inclusion of 200 patients would result in a ratio of ∼20:1, which was considered abundant [26, 27].

## Results

### Feasibility and face validity—Step 1

We received 24 responses: 14 (58.3%) females and 10 (41.7%) males, with a mean age of 63.5 (8.0) years. The educational levels of the participants were junior high school/basic education *n* = 3 (12.5%), high school/vocational training *n* = 14 (58.3%) and university/college *n* = 7 (29.2%). An average of 14.0 min to fill in the questionnaire was reported from 23 participants.

In addition to answering the P3CEQ, participants answered additional questions concerning understandability, response options, relevance and usefulness of instructions, as well as acceptability of questions. Five respondents found questions hard to understand, with comments reflecting the consequence of poor wording and improper terminology. Two reported not finding an appropriate response option, with unfamiliar terms being the cause. Twenty-two responded to the question about relevance, and 16 (72.7% of these) found questions in items relevant. The remaining showed comments that reflected vague questions or improper language. None found the questions disrespectful or offensive.

According to feedback from user representatives, and the results of the feasibility study, the translated version of the P3CEQ was adjusted linguistically to better suit the Norwegian healthcare terminology and context.

### Patient characteristics—Step 2

In Step 2, we received 181 responses: 90 from the local hospital and 91 from the university hospital. However, five responses (three local and two university hospitals) were excluded due to insufficient data. The documented number of patients declining participation was 62, 33 (53.2%) women and 29 (46.7%) men, with a mean age of 69.7 (10.8) years. Reasons for declining included not wanting to participate and feeling too frail, among others. Respondents’ demographic characteristics are presented in Table 1.

**Table 1:** Self-reported participant demographics in P3CEQ validation

*N* = 176 (100)	
Age group (years)	*n* (%)
≤40	3 (1.7)
41–50	9 (5.1)
51–60	34 (19.3)
61–70	70 (39.8)
71–80	55 (31.5)
≥81	5 (2.8)
Gender	*n* (%)
Female	92 (52.3)
Male	84 (47.7)
Marital status	*n* (%)
Single	45 (25.6)
Married/partner	131 (74.4)
Living arrangements	*n* (%)
Alone	43 (24.4)
With others	133 (75.6)
Education [missing] [4] (2.3%)	*n* (%)
Primary or junior high school	33 (19.2)
Vocational education/high school	79 (45.9)
College/university	60 (34.9)

Participants’ age ranged from 29 to 91 years and was skewed towards an elderly population. The median age was 67.0 years (25pc = 60 and 75pc = 73), and 102 (58.0%) were aged ≥65 years. Out of the participants who lived with more persons than their partner, 16 reported to live with children and one with others.

The most frequent types of cancer were breast *n* = 68 (38.6%), followed by prostate *n* = 55 (31.3%), lung *n* = 15 (8.5%) and others *n* = 38 (21.6%).

Details about participants’ treatments are described in Table 2. The number of treatment sessions ranged from 2 to 44, with a median of 15.0 treatments (25pc = 15 and 75pc = 30). The duration of treatment (days from start to completion) ranged from 1 to 81 days, with a mean of 27.2 days (SD 15.2). The intent of treatment was curative in 143 (81.3%) and palliative in 33 (18.8%) of the total cases.

**Table 2:** Treatment information from medical records

Radiotherapy sessions	*N* (%)
≤5	11 (6.3)
6–15	84 (47.7)
16–25	29 (16.5)
26–35	48 (27.3)
≥36	4 (2.3)
Treatment period (days)	*N* (%)
≤6	7 (4.0)
7–14	21 (11.9)
15–21	60 (34.1)
22–28	24 (13.6)
29–35	13 (7.4)
36–42	9 (5.1)
≥43	42 (23.9)
Previously received radiotherapy	*N* (%)
Yes	27 (15.3)
No	149 (84.7)

### Response distributions

An overview of the responses to each item is displayed in Table 3. Likert-scale items with a ceiling effect >50% (‘best score’) were Item 3 (considered ‘whole person’) (54.1%), Item 4 (patients compelled to repeat health record information to healthcare professionals) (69.0%), Item 9 (information to self-manage) (53.5%) and Item 10 (confidence to self-manage) (60.8%). Items 7b–d are not included, due to insufficient data.

**Table 3:** P3CEQ responses

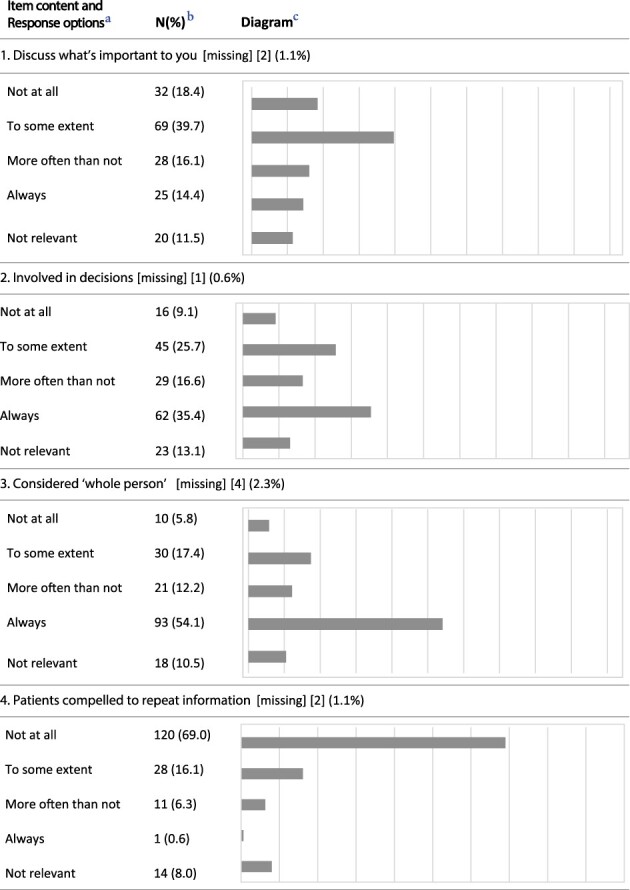
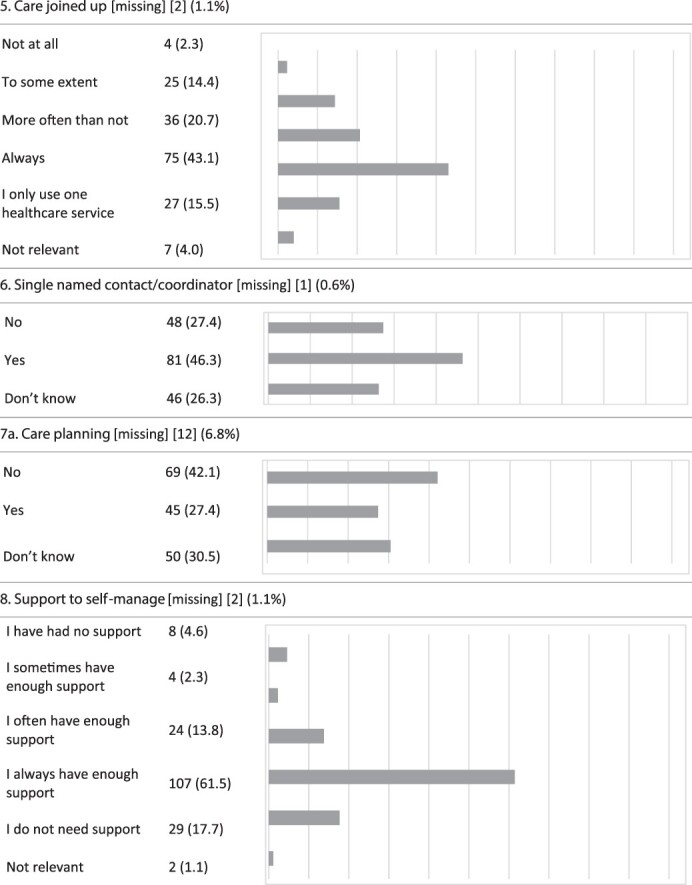
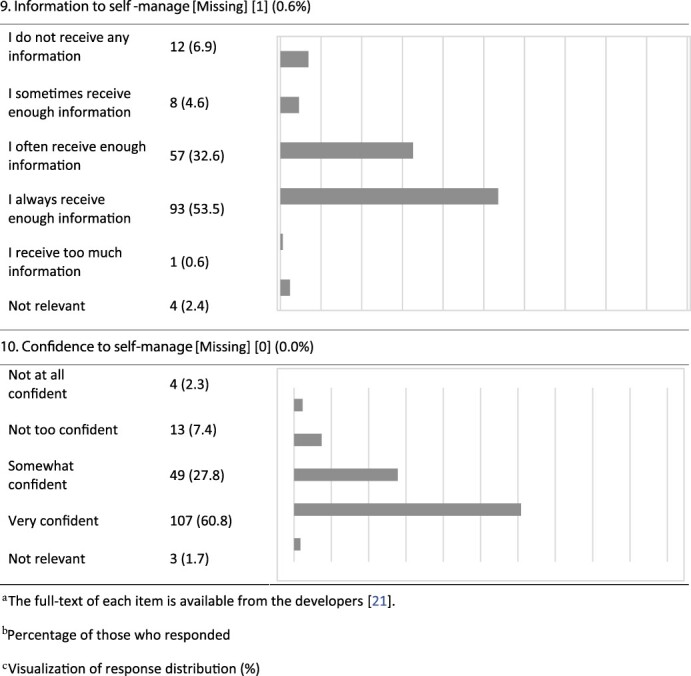

### Construct validity

Three factors in the measure were determined by the exploratory factor analysis (Table 4). Each item loaded >0.4, which is considered a sufficient commonality in such data [28, 31].

**Table 4:** Exploratory factor analysis (varimax rotation) of P3CEQ items

Rotated component matrix			
Item content	Person-centredness	Support	Service coordination
1. Discuss what’s important to you	**0.773**	0.004	0.053
2. Involved in decisions	**0.832**	−0.065	0.019
3. Considered ‘whole person’	**0.685**	0.201	0.277
4. Patients compelled to repeat information	0.227	0.336	**0.447**
5. Care joined up	0.366	**0.520**	0.255
6. Single named contact/coordinator	0.115	−0.182	**0.862**
7. Care planning (mean)	0.057	0.378	**0.623**
8. Support to self-manage	−0.010	**0.673**	0.067
9. Information to self-manage	0.025	**0.537**	0.303
10. Confidence to self-manage	−0.013	**0.765**	−0.113

Bold values show which variables are most strongly correlated (highest loading) with each factor.

### Internal consistency

For the Person-centredness scale, Cronbach’s *α* = 0.714. For the Service coordination scale, Cronbach’s *α* = 0.485 when including the items identified through the rotated component matrix (Table 4). When removing Item 4, Cronbach’s *α* increased to 0.511. Thus, we tested Item 4 in the ‘Support’ scale, where it loaded 0.336 in the factor analysis. For the Support scale, Cronbach’s *α* = 0.611 when including the items identified through the rotated component matrix (Table 4). When including Item 4 in the Support scale, Cronbach’s *α* increased to 0.649.

For all 10 items, Cronbach’s *α* = 0.698.

## Discussion

### Statement of principal findings

The main findings show that the questions were relevant, but an adjustment in terminology and linguistic features was required. Our factor analysis (construct validity) identified three distinct sub-scales. This differs from the validity test of the English version, where the factor analysis revealed two factors [21]. The internal consistency for the selected three factors differed between Cronbach’s *α* = 0.511 and 0.714, which is lower than in the initial validation [21]. Four items (3, 4, 9 and 10) had ceiling effects >50%.

### Strengths and limitations

One strength of this study is the variety within the sample. Our study covers the most relevant groups of patients in a radiotherapy setting. Curative and palliative treatments, as well as different cancers and different treatment spans, are represented, all of which reflect the general population of patients with cancer receiving radiotherapy [2]. The diversity in patients’ diagnosis and treatment intent may have influenced the results. However, the P3CEQ is designed to measure patients’ experiences with services (PREM), which may be modestly influenced by these factors [23]. A tool that covers all patients receiving radiotherapy in the improvement of services is crucial.

It may be argued that exploratory factor analysis by applying principal component analysis is not the most modern method to find structures in data sets. However, it is a widely used method, and in the validation of both the UK and Dutch versions of the P3CEQ [21, 23], the same method was used. Thus, in a new setting, we consider exploratory factor analysis to be the best method to compare the different versions.

Furthermore, a test–retest to confirm the reliability of the scale would have been desirable. This was not feasible. The P3CEQ was distributed to patients at the end of treatment and returned in a pre-paid envelope. This is also a strength, as patients could fill in their responses without interference from healthcare providers.

Items 3 and 7a had the most missing answers, which may indicate a need for reconsideration of these items [26]. Item 3 (considered ‘whole person’) may be complex and hard to understand. Additionally, when receiving services from multiple providers, this may be difficult to evaluate. Considering Item 7a (care planning), an explanation for the high missing rate may be that participants are unaware of the structuring and arrangements involving the services they receive [5].

### Interpretation within the context of the wider literature

We chose the P3CEQ as a potentially suitable PREM to assess patients’ experiences in a radiotherapy setting. The choice was made based on results from a preceding qualitative study [13], which supported the measure’s relevance in the population where it was applied. This approach is supported by Male *et al*. [16], who described the development of PREMs using qualitative data to elucidate concepts.

The face validity of the questionnaire was assessed through our feasibility and face validity study (Step 1). We consider the linguistic adjustments to improve feasibility. Additionally, the initial P3CEQ validation study [21] based face validity on a literature review and workshops with stakeholders [14]. The constructs revealed through our factor analysis coincide with areas of importance to older adults with cancer [13]. As most patients with cancer are aged ≥65 years [1, 2], we consider the constructs found highly applicable as 102 (58.0%) of participants in this study were aged ≥65 years. Furthermore, this study has low levels of missing data (displayed in Table 3), which supports good face validity according to Polit and Yang [26]. Considering these arguments, we find the Norwegian version of the P3CEQ feasible, acceptable and, for most of the patients who tested the scale, relevant. Consequently, we consider the face validity to be good.

Lloyd *et al*. [21] found two factors through their factor analysis: Person-centredness (Items 1–5 and 8–10) and Care-coordination (Items 6 and 7). A recent Dutch study also found two factors [23], which is quite similar to Lloyd *et al*. [21]. In our study, we found a Person-centredness factor (Items 1–3) and a Service coordination factor (Items 4, 6 and 7), and the remaining four items in our analysis clearly formed a *third* group, which we named ‘Support’ (Items 5 and 8–10). The three items in our Person-centredness factor are about how one is met as a person, while the five items in our ‘Support’ factor are about practical approaches such as information, help and self-management. Items 6 and 7 probe planning and coordination. All items in our study had a strong loading to their respective group (>0.500), except for Item 4, loading 0.447, which is also considered sufficient [27, 28]. However, we chose to move Item 4 to the ‘Support’ factor on the basis of assessing internal consistency. An explanation for different findings from Lloyd *et al*. [21] and Rijken *et al*. [23] may be differences in healthcare services in different countries. The UK and Dutch samples consist of adults with long-term conditions and chronic conditions, respectively, whereas the present study included patients in active treatment over a shorter period of time. This may have influenced responses, especially on Item 7a (care planning), where over 30% responded ‘Don’t know’, resulting in low scores independent of responses to Items 7b–d. Additionally, different settings (i.e. frequent users of health care [21] and radiotherapy in specialist care) may also be an explanation. Considering our findings and difference in care settings, we have decided to proceed with three factors in the Norwegian version of the P3CEQ.

Our discovery of a lower Cronbach’s *α* compared to the initial validation [21] may be caused by the differences in samples, i.e. lower *N* and a lower number of items in our factors [26, 29]. Although the sample size is considered sufficient [26, 27], one would ideally wish for more participants. The Person-centredness scale had a Cronbach’s *α* of >0.7, which is considered acceptable [26]. Both the Service coordination scale and the Support scale were between 0.5 and 0.7, which are in the lower bounds, but the overall scale had Cronbach’s *α* = 0.698, which can be considered sufficient [30]. Considering that all items contribute to the scale, the scale—with its current 10 items—remains.

A weakness in the items with ceiling effects (3, 4, 9 and 10) may be their insufficient ability to detect change over time (e.g. improvements in scores) [26]. However, we consider these four items important as the P3CEQ provides experiences from patients with complex, protracted care needs, consistent with the radiotherapy setting [6], and thus the items should be kept. With the three factors identified, the scale allows for distinction between different areas in the cancer care pathway.

### Implications for policy, practice and research

We have found three distinct factors in our analysis, consistent with areas of importance for older patients receiving radiotherapy. The factors ‘Person-centredness’, ‘Support’ and ‘Service coordination’ can provide insight into the different areas of patients’ experiences of radiotherapy. We have not identified any specific measures to record patients’ experiences of the entire radiotherapy pathway, and a valid and reliable tool is crucial to assessing and thus improving services.

## Conclusions

This is a Norwegian study in a Norwegian healthcare context, and conclusions must consider this. Nevertheless, in the development of PREMs in general and P3CEQ especially, experiences from different translations (versions) are valuable.

The P3CEQ is an applicable PREM for further use to evaluate the complex services offered to cancer patients receiving radiotherapy. We have established that the Norwegian version of the P3CEQ is a relevant, valid and reliable tool for patients receiving radiotherapy. However, further testing on a larger sample is necessary to confirm the factors detected in the present study.

## Data Availability

The data underlying this article cannot be shared publicly due to participants not consenting to sharing of data. The data will be shared on reasonable request to the corresponding author.

## Abbreviations

25th and 75th percentiles - 25pc and 75pc

P3CEQ - Person-centred coordinated care experience questionnaire

PC - Person-centeredness

PCA - Principle component analysis

PCC - Person-centred care

PREM - Patient-reported experience measure
